# The Sociodemographic Benefits of Extending MS‐2Step to 70 Days in Australia

**DOI:** 10.1111/ajo.70077

**Published:** 2025-11-21

**Authors:** Laura Slade, Jennie Louise, Katina D'Onise, Jodie Dodd

**Affiliations:** ^1^ Women's and Children's Hospital North Adelaide South Australia Australia; ^2^ Robinson Research Institute University of Adelaide Adelaide South Australia Australia; ^3^ Women's and Children's Hospital Research Institute Women's and Children's Hospital North Adelaide South Australia Australia; ^4^ South Australian Health and Medical Research Institute Adelaide South Australia Australia; ^5^ School of Public Health University of Adelaide Adelaide South Australia Australia

**Keywords:** abortion access, maternal health, termination of pregnancy

## Abstract

In many areas of the world, outpatient early medical abortion (EMA) is provided through mifepristone and misoprostol up to 10 weeks or 70 days gestation; however in Australia access is restricted to 63 days. A retrospective cohort study using South Australian data from 2012 to 2020 compares women undergoing abortion at less than 9 weeks with women undergoing abortion at less than 10 weeks. Currently, socioeconomic disadvantage is associated with a higher rate of presenting for abortion after 9 weeks. Extending EMA access would be of particular benefit for groups with socioeconomic disadvantage.

## 1 Introduction

Early medical abortion (EMA) is performed most safely and effectively with a combination of mifepristone, a progesterone antagonist, and misoprostol, a prostaglandin analogue. Across the world, these medications are used in outpatient settings with high efficacy for women seeking early pregnancy abortion [1, 2]. Costs vary by country, but in many settings, EMA is cheaper than a surgical alternative for abortion [3]. The flexibility of these medications also minimises associated costs for travel and accommodation by postal delivery to those in rural and remote areas, as well as meaning the medication can be taken at a time convenient to the individual's circumstances, rather than being dictated by a clinic schedule.

In South Australia, the combination of mifepristone and misoprostol is marketed as ‘MS2‐Step’. Access to EMA and mifepristone in Australia has been a lengthy and politicised battle [4]. Although mifepristone has been safely used across the world for decades, Australia was one of the last high‐income countries to permit the importation of mifepristone in 2012. Initially this was only until 49 days' gestation but extended to 63 days' gestation in 2015 [5]. While the Therapeutic Goods Administration (TGA) regulates access to EMA, these regulations have hampered access to this medication. Although mifepristone can be prescribed outside of this gestational age limit, often practitioners are reluctant to do so. The TGA regulations also govern when the medication will be subsidised on the pharmaceutical benefits scheme which reduces the cost under Medicare by over 10 fold.

Abortion is now legalised in all states and territories of Australia; however the regulations governing EMA have continued to restrict access. The regulations, until August of 2023, mandated additional training for medical providers who were required to become authorised prescribers. Other prescribers including nurse practitioners and endorsed midwives were unable to prescribe mifepristone. Pharmacists were also required to have additional training to dispense mifepristone [6]. Until 2021 in South Australia, community pharmacies were unable to dispense mifepristone because of the legal requirement for abortion to begin within a medical facility. The removal of these restrictions now means mifepristone can be prescribed like any other medication for outpatient EMA prior to 63 days' gestation.

In South Australia women in rural areas, who are teenagers and who live in areas of socioeconomic disadvantage are all less likely to access abortion at a gestation eligible for EMA [7]. Extending the gestational limit on access to EMA is one strategy to improve access. The Royal Australian and New Zealand College of Obstetricians and Gynaecologists (RANZCOG) Clinical Guideline for Abortion Care provides the local guideline for EMA up to 10 weeks gestation [8], however the use of EMA in this way would be subject to changes in TGA approval. The aim of this study was to assess the impact of extending eligibility for outpatient EMA in Australia to 70 days on the proportion of subgroups of women who would be eligible compared to ineligible at the time of abortion.

## 2 Methods

Since 1970, South Australian law has mandated the recording of all abortions to a centralised register. This is completed at the time of the abortion by the performing/prescribing clinician and includes basic demographic information including age, postcode, parity, gestation and indication for abortion. A retrospective cohort study was conducted using data from the registry from 2012 to 2020 when EMA has been available in South Australia to compare women undergoing abortion at less than 9 weeks with women undergoing abortion at less than 10 weeks. Teenage was defined as 19 or fewer years of age at the time of abortion. Postcodes were coded according to the 2016 Australian Statistical Geographical Classification Remoteness Area (AGSC‐RA) system and the Australian Bureau of Statistics 2018 Index of Relative Socio‐Economic Advantage and Disadvantage (IRSAD). The data were compared descriptively.

Ethics approval was obtained from the Department of Health, South Australia, which is the data custodian for the registry, with reference number 2022/HRE00176.

## 3 Results

Between 2012 and 2020 a total of 39 338 women have undergone abortion in South Australia. Using the current threshold of < 9 weeks, 71.0% of women undergo abortion at a gestation eligible for EMA. Increasing this threshold to < 10 weeks would increase the proportion of women eligible to 79.0% (Table 1). The average age is not different between thresholds; however increasing the gestational limit to < 70 days would increase the proportion of teenagers eligible from 62.1% to 70.9%. Parity was poorly recorded across this time, but the proportion of nulliparous women would also increase.

**TABLE 1:** Demographics of women by gestation at abortion (*n*, % of total for all women).

	< 9 weeks	< 10 weeks	All women who had an abortion
*Total*	27 920 (71.0%)	31 080 (79.0%)	39 338
Age (years) (median (IQR))	25 (21, 32)	25 (21, 31)	
Teenage *n* (%)	2607 (62.1%)	2974 (70.9%)	4196
Nulliparous	4187 (71.5%)	4637 (79.2%)	5856
Missing parity *n* (%)^a^	9311 (33.4%)	14 024 (45.1%)	12 930 (32.9%)
*Remoteness* ^b^			
Major city	20 299 (72.7%)	22 479 (80.3%)	27 992
Inner regional	5045 (68.6%)	5648 (76.9%)	7349
Outer regional	1657 (64.8%)	1892 (73.9%)	2559
Remote	781 (67.1%)	892 (76.6%)	1164
Very remote	107 (54.9%)	133 (68.2%)	195
*Index of relative socio‐economic advantage and disadvantage* ^b^			
Q1	8335 (64.8%)	9535 (74.2%)	12 854
Q2	3885 (69.6%)	4342 (77.7%)	5585
Q3	5381 (72.8%)	5947 (80.5%)	7391
Q4	6961 (75.7%)	7612 (82.8%)	9190
Q5	3316 (78.0%)	3596 (84.5%)	4254

Abbreviation: IQR, interquartile range.

^a^ % is the percentage of total for each subgroup of gestation (row percentages).

^b^ Missing data accounts for < 1% of total.

The highest proportion of women who are eligible, irrespective of the threshold, is from major cities, with only 54.9% of women in very remote areas currently undergoing abortion at a gestation eligible for EMA. However, those in remote and very remote areas have the highest proportional increase with a gestational limit of < 70 days, with the proportion eligible increasing from 67.1% to 76.6% for women in remote areas and 54.9% to 68.2% for women in very remote areas. The proportion of women eligible in quintile 1 increased from 64.8% to 74.2% with the change in gestational threshold; however this remains 10% behind quintile 5 where 84.5% of women would be eligible at a threshold of < 10 weeks.

## 4 Discussion

This study highlights the important socio‐demographic benefits of extending EMA access in Australia. Socio‐economic disadvantage is strongly correlated with the gestation at abortion [7]. Although this analysis could not explain the specific reasons associated with accessing abortion at a later gestational age, travel and clinic accessibility are commonly attributed [9]. Importantly, health literacy is often attributed as a cause of later diagnosis of pregnancy; however studies from the United States have highlighted that this is a minor barrier in comparison to cost and travel logistics when accessing abortion care [10].

Rurality in Australia is a consistent barrier to access abortion services with limited practitioners offering services [11, 12]. Even when a practitioner is available, services may be intermittent or women may choose to avoid their local service to preserve their anonymity [13]. Although telehealth services are often thought of as the solution for remote areas, anyone living more than 2 h from 24‐h emergency care has historically been ineligible for telehealth EMA [14] and this cannot be seen as the only solution to improving access.

Extending MS2‐Step access would likely benefit access to abortion services as women have more time to present for care. Although cost is a common barrier, at the time of this study, public abortion clinics were running in the major urban areas with low IRSAD rankings [15]. These clinics offered government‐funded services for those who were eligible, meaning out‐of‐pocket costs were minimal. Presumably, this indicates that there are access barriers beyond cost. Extending the gestational age of eligibility is a simple approach to broaden access.

There is a large body of literature supporting the use of mifepristone and misoprostol in combination for EMA up to 10 weeks gestation, as is currently used in many countries, across the world. The United States of America, New Zealand and the United Kingdom all permit the use of mifepristone at home for EMA up to 70 days gestation [16, 17]. Canada, like many of these countries has allowed mifepristone use at progressively higher gestations over time without a significant change in the rate of complications or the total abortion rate [18]. The World Health Organisation (WHO) recommends that the same EMA medications can be safely used up to 12 weeks gestation, although it should be noted that the quality of evidence supporting use to this gestation is low [19].

Comparisons of EMA with mifepristone and misoprostol between 64 and 70 days gestation compared with 63 or fewer days have shown relatively similar efficacy, acceptability and side effect profiles [20, 21, 22]. Importantly, a consistently high level of acceptability for women is reported. There is very limited information comparing EMA to surgical abortion in the late first trimester; however the available data does not suggest a significant difference in efficacy or complication rates overall [23]. The only significant difference seen was higher rates of nausea in the EMA group compared with the surgical abortion group [24].

Importantly, this study only includes the women who were ultimately able to access abortion services and not those who instead continued with their pregnancy. Specific information about factors that could explain the differences in access seen was not available from this dataset and should be the subject of more research.

## 5 Conclusion

Extending the approval of MS2‐Step in Australia would align with the use of these medications in many areas of the developed world. This could have particular benefits for groups with socioeconomic disadvantage, teenagers and those in rural areas who have higher rates of undergoing abortion at a gestation above the current threshold of 63 days' gestation.

## Conflicts of Interest

The authors declare no conflicts of interest.
